# The Impact of Model Building on the Transmission Dynamics under Vaccination: Observable (Symptom-Based) versus Unobservable (Contagiousness-Dependent) Approaches

**DOI:** 10.1371/journal.pone.0062062

**Published:** 2013-04-12

**Authors:** Keisuke Ejima, Kazuyuki Aihara, Hiroshi Nishiura

**Affiliations:** 1 School of Public Health, The University of Hong Kong, Hong Kong SAR, China; 2 Department of Mathematical Informatics, Graduate School of Information Science and Technology, The University of Tokyo, Tokyo, Japan; 3 Institute of Industrial Science, The University of Tokyo, Tokyo, Japan; 4 PRESTO, Japan Science and Technology Agency, Saitama, Japan; Harvard School of Public Health, United States of America

## Abstract

**Background:**

The way we formulate a mathematical model of an infectious disease to capture symptomatic and asymptomatic transmission can greatly influence the likely effectiveness of vaccination in the presence of vaccine effect for preventing clinical illness. The present study aims to assess the impact of model building strategy on the epidemic threshold under vaccination.

**Methodology/Principal Findings:**

We consider two different types of mathematical models, one based on observable variables including symptom onset and recovery from clinical illness (hereafter, the “observable model”) and the other based on unobservable information of infection event and infectiousness (the “unobservable model”). By imposing a number of modifying assumptions to the observable model, we let it mimic the unobservable model, identifying that the two models are fully consistent only when the incubation period is identical to the latent period and when there is no pre-symptomatic transmission. We also computed the reproduction numbers with and without vaccination, demonstrating that the data generating process of vaccine-induced reduction in symptomatic illness is consistent with the observable model only and examining how the effective reproduction number is differently calculated by two models.

**Conclusions:**

To explicitly incorporate the vaccine effect in reducing the risk of symptomatic illness into the model, it is fruitful to employ a model that directly accounts for disease progression. More modeling studies based on observable epidemiological information are called for.

## Introduction

There are two intriguing characteristics in quantitatively modeling infectious disease data. First, the risk of infection to an individual is dependent on the risks of other individuals in the same population unit. Second, the infection event is seldom directly observable. Among these two, the dependence has been addressed during the process of model building, e.g., a heterogeneous contact structure has been explicitly considered in various types of models [1] and sometimes by examining the conditional risk of infection at a confined setting (e.g. household). On the other hand, it has been common to address the unobservable nature of infection event by employing a convolution equation, i.e. the so-called “backcalculation method”, to infer the time of infection based on the dataset of illness onset [2]–[5]. However, the deconvolution procedure has been frequently dealt with as a statistical technique that is independent of the transmission model [6], and the process of model building tended to be separated from the unobservable character of infection event.

Ignoring the unobservable nature during model formulation would complicate the model fitting to empirical data. In many instances, a temporal distribution of infected individuals (i.e. an epidemic curve) is analyzed, and most frequently, the best available dataset is the daily counts of cases. The data are usually collected based on observable information only, e.g. counts of cases according to the date of diagnosis of clinically apparent illness. Only in the better case, epidemiologists are granted an access to the daily frequency of illness onset. Nevertheless, the data generating process of the empirical information is rather different from assumed transition mechanism within the so-called SIR (susceptible-infectious-removed) model. The SIR model is considered as inconsistent with the data, because the transition from S to I state is determined by the event of infection (which is unobservable) and the other transition from I to R state is determined by the loss of infectiousness (which is even more difficult to observe) [7]. In light of a need to construct a model that better adheres to the observable information, a previous study proposed a novel modeling approach that classifies infected individuals into asymptomatic and symptomatic ones while still adopting a common multistate model structure [8]. In the case of the unobservable SEIR (susceptible-exposed-infectious-removed) model, the model handles unobservable information within the multistate structure, classifying infected individuals into pre-infectious (exposed) and infectious individuals [7], [8] that are not directly distinguishable from each other in empirical observation.

Although a previous study recognized the importance of asymptomatic transmission in considering the feasibility of non-pharmaceutical public health interventions (e.g. contact tracing and case isolation) [9], the impact of correctly and precisely capturing the natural course of “illness” on the effectiveness of interventions (e.g. vaccination) has yet to be discussed. In the past, the contribution of asymptomatic individuals to the transmission dynamics tended to be modeled by employing the widely adopted SEIR model while splitting infectious individuals (I-class) into symptomatic and asymptomatic cases (e.g. [10]). The underlying assumptions and any potential drawbacks for employing the SEIR model on this matter have not been clarified, and thus, we would like to examine if an epidemic threshold (which yields the critical vaccination coverage) is greatly influenced by the abovementioned difference in model building approaches.

Employing a mathematical modeling approach, the present study aims to assess the impact of model building strategy on the transmission dynamics of an infectious disease under vaccination practice. In particular, we investigate differential values of epidemic threshold between models that rest on observable and unobservable information.

## Materials and Methods

### Two models

**Figure 1:**
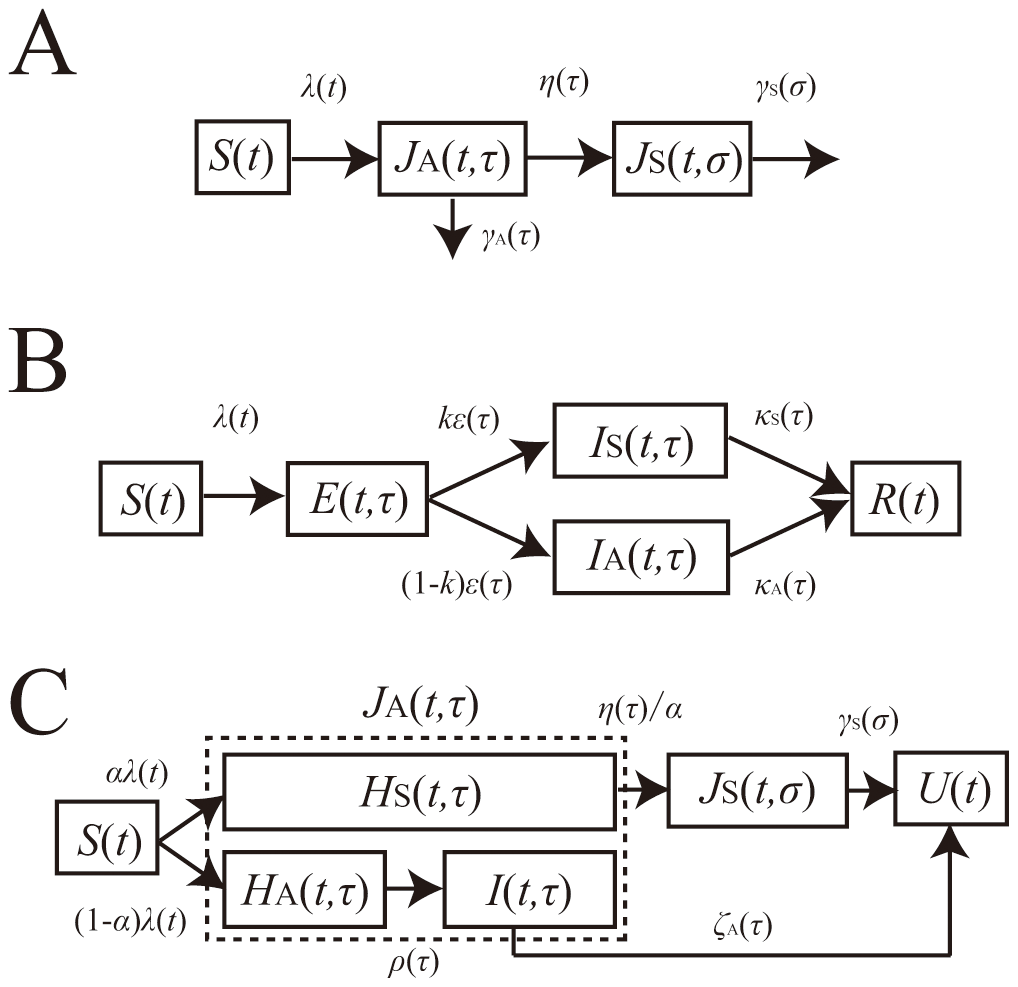
Compartments of observable and unobservable models. A. The compartment of an observable model. The model describes the transitions depending on illness onset and recovery from clinical symptoms. Once infected, all infected individuals experience asymptomatic period, *J*_A_, some of which fully recover from infection without symptoms, and the remaining develop symptoms, *J*_S_. B. The compartment of an unobservable model. The model describes the transitions depending on acquirement or disappearance of infectiousness. Upon infection, infected individuals experience the latent period (i.e. Exposed compartment (*E*)) after which each acquires infectiousness and is classified as either symptomatic (*I*_S_) or asymptomatic (*I*_A_) one. C. The compartment of the special case of the observable model. The model describes the transitions based on symptoms, but partially accounts for infectiousness too. To let it be similar to model B, we decomposed asymptomatic individuals, *J*_A_ of the observable model (panel A) into pre-symptomatic individuals, *H*_S_ and fully asymptomatic individuals with or without infectivity. *U* represents recovered individuals.

We consider two different types of mathematical models, one based on observable variables including symptom onset and recovery from clinical illness (hereafter referred to as the “observable model”) and the other based on unobservable information including infection event and infectiousness (the “unobservable model”). Whereas the unobservable model in the following is a variant of the SEIR model [10], the observable model considers the transition of infected individuals based on illness onset and the disappearance of symptoms that are directly visible in the field data [8] (Figure 1A and 1B). The word “observable” is intended to reflect the presence of observable symptoms (i.e. not including those observed or detected by employing laboratory testing during the asymptomatic period). Thus, the observable model might also be referred to the “symptom-based” model. Similarly, the unobservable model may be referred to as the “contagiousness-dependent” model.

Here we briefly describe the time-dependent growth of an epidemic based on the observable model, the compartments of which are drawn in Figure 1A. Let *J*_A_(*t*,*τ*) and *J*_S_(*t*,*σ*) be the numbers of asymptomatic and symptomatic cases at calendar time *t*, infection-age *τ* since infection and disease-age *σ* since illness onset. The growth of cases is described by:
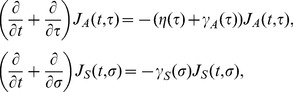
(1)


where *η*(*τ*) is the rate at which asymptomatic cases develop symptoms, and *γ*_A_(*τ*) and *γ*_S_(*σ*) are the rates at which asymptomatic and symptomatic cases are fully recovered. We consider an initial growth phase of an epidemic at which the depletion of susceptible individuals *S*_0_ is negligible. Let *λ*(*t*) be the force of infection, or the rate at which susceptible individuals are infected. Two boundary conditions, i.e., the new infection and new illness onset, are written as
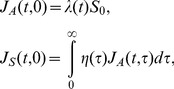
(2)


where *λ*(*t*) is, by adopting a mass action principle, parameterized as:
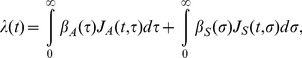
(3)


where *β*_A_(*τ*) and *β*_S_(*σ*) are the infection-age and disease-age dependent rates of secondary transmission, respectively. It should be noted that the recovered individuals in Figure 1A are assumed as no longer infectious. An advantage of this modeling approach is that a reasonable computation of epidemiological measurements (e.g. the reproduction number, the generation time and the serial interval) can be achieved, adhering to observed available information [8]. Moreover, transitions from the asymptomatic state to the symptomatic or recovered state are in line with the actual clinical course of infection, i.e., only a part of asymptomatic individuals develop symptoms and the rest of infected individuals recover from infection without symptoms.

The basic reproduction number of this model is computed as follows ([8]):
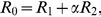
(4)


where *R*_1_, *R*_2_ and *α* are the average number of secondary cases generated by a single asymptomatic case (only during the asymptomatic period), the average number of secondary cases generated by a single symptomatic case throughout the course of the symptomatic period, and the conditional probability of developing symptom given infection, respectively. The probability of symptomatic illness, *α* is multiplied to *R*_2_ only, because all infected individuals experience asymptomatic class while only the fraction *α* of infected individuals result in symptomatic infection. The model (1) is a stage-structured model in which the reproduction number is calculated from the integral kernel of the specific class of host in its renewal equation [11]. *R*_1_, *R*_2_ and *α* are defined as
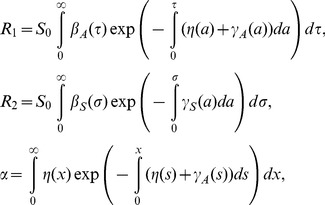
(5)


which we will use in later discussion.

The other type of a model, i.e., the unobservable model, can be said to be the infection-age structured SEIR model that further classifies infectious individuals into symptomatic and asymptomatic cases [10] (Figure 1B). Let *E*(*t*,*τ*), *I*_A_(*t*,*τ*,*σ*) and *I*_S_(*t*,*τ*,*σ*) be the numbers of pre-infectious individuals, asymptomatic infectious individuals and symptomatic infectious individuals, respectively, at calendar time *t*, infection-age *τ* and disease-age *σ*. The dynamics is described by
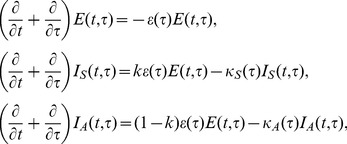
(6)


where *ε*(*τ*), *κ*_A_(*τ*) and *κ*_S_(*τ*) represent the rate of acquiring infectiousness, and the recovery rates among asymptomatic and symptomatic infectious individuals, respectively. *k* is the weight (0≤*k*≤1) of the rate at which exposed individuals acquire infectiousness that determines the probability of developing symptom. A boundary condition for new infections is
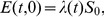
(7)


where the force of infection is
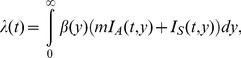
(8)


where *β*(*τ*) represents the rate of secondary transmission at infection-age *τ*, and *m* represents the relative infectiousness of asymptomatic cases as compared to symptomatic cases. The basic reproduction number, *R*_0_, for this unobservable model is given by
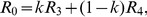
(9)


where *R*_3_ and *R*_4_ are the average numbers of secondary cases generated by a single asymptomatic case and a single symptomatic case throughout the course of infectiousness, respectively. In equation (9), *k* and (1−*k*) are multiplied to *R*_3_ and *R*_4_, respectively, because the probabilities of an infected individual to experience symptomatic and asymptomatic infections are given by *k* and (1−*k*), respectively. Again, the reproduction numbers, *R*_3_ and *R*_4_, are calculated from the integral kernel of the renewal process, i.e., we define
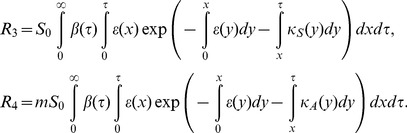
(10)


Using these two models under a homogeneously mixing assumption, we investigate the importance of appropriately capturing the observable natural course of infection in epidemiological models.

### Analytical and numerical analyses

To explicitly account for the observable clinical course of infection, underlying assumptions of using a parameter *k* in the unobservable model as the probability of symptomatic infection remain unclear (Figure 1B and system (6); because the transition from E to I state does not have anything to do with illness onset). Moreover, it is fruitful to identify different model assumptions between two models and their practical relevance to infectious disease control. Thus, here we take two different approaches to identify the structural differences and different assumptions between two models. First, we impose additional assumptions to the observable model, thereby permitting it to resemble the SEIR-like unobservable model. A simplistic analytical computation is performed to mathematically determine the difference between the two models. Second, we numerically compute the basic reproduction numbers based on the two models. It is clear, even intuitively, that the presence of pre-symptomatic transmission is a major difference between the observable model and the unobservable SEIR type model. Thus, we examine the sensitivity of the basic reproduction number to the proportion of pre-symptomatic secondary transmissions among the total of asymptomatic transmissions.

Subsequently, we investigate the differential impact of vaccination on the reproduction number (or, on the epidemic threshold) of the two models. In a published study, the next-generation matrix was employed to incorporate various different biological actions of vaccination into the transmission dynamics under vaccination [12]. However, the derivation of the next-generation matrix in the published study remained heuristic, and moreover, the computation rested only on the unobservable SEIR-like model. Thus, here we derive the next-generation matrix based on the linearized system of both (1) and (6), measuring the impact of differential model formulation on the reproduction number. When analytically computing the matrix, various different effects of vaccination are considered, including not only the reductions in susceptibility and infectiousness but also the reduction in the risk of symptomatic illness [13].

### Parameter values

For numerical illustration, we examine the plausible parameter space for four different viral infectious diseases. Table 1 shows the parameter values that are adopted to numerically calculate the threshold quantities and other associated variables of observable and unobservable models [8]–[10], [12], [14]–[24]. Smallpox is considered for the exposition of the similarity between two different models, because it involves very few asymptomatic transmissions [14], [25], [26]. HIV/AIDS is the opposite example of smallpox with respect to the proportion of asymptomatic transmissions among the total of secondary transmissions. Namely, the secondary transmission mostly occurs before the onset of AIDS [8]. Influenza and varicella are considered as examples that lie between smallpox and HIV/AIDS. In particular, influenza is considered, because (i) the unobservable model with asymptomatic and symptomatic infectious individuals was initially employed with an application to influenza [10] and (ii) a variety of vaccine effects have been quantified based on challenge and community-based studies [12], which offers a suitable condition to explore the impact of model formulation on the transmission dynamics in the presence of vaccination. It should be noted that successful vaccine of HIV has yet to be offered [27] and the corresponding vaccine effect parameters were only hypothetically assumed.

**Table 1:** Parameter values for observable and unobservable models of directly transmitted infectious diseases.

Description	Notation	Parameter values				References/Assumptions
		Smallpox	Influenza	HIV	Varicella	
The average number of secondary cases produced by an asymptomatic case	*R*_1_	0.69	0.60	3.67	3.24	[14] & calculated
The average number of secondary cases produced by a symptomatic infection	*R*_2_	6.18	1.20	0.00	3.24	[14] & calculated
The average number of secondary cases produced by a fully asymptomatic case	*R*_a_	1.37	0.96	6.12	6.47	[14] & calculated
Probability of developing symptoms in the unobservable model	*α* (or *k*)	1.00	0.75	0.80	1.00	[10] & assumed
Basic reproduction number of the observable model	*R*_0_	6.87	1.50	3.67	6.47	[14], [15], [16], [17], [18]
Proportion of asymptomatic transmissions among all secondary transmissions	*θ*	0.10	0.40	1.00	0.50	[8], [9], [21], [22]
Proportion of pre-symptomatic transmissions among all asymptomatic infection	*g*	1.00	0.60	0.67	1.00	[23] & calculated
Vaccine efficacy of reducing infectiousness	VE_I_	0.80	0.15	0.60	0.80	[12], [17], [19], [20], [21], [22], [24] & assumed
Vaccine efficacy of reducing susceptibility	VE_S_	0.95	0.41	0.40	0.50	[12], [17], [19], [20], [21], [22], [24] & assumed
Vaccine efficacy of preventing progression to symptomatic illness	VE_P_	0.87	0.67	0.60	0.50†	[12], [17], [19], [20], [21], [22], [24] & assumed

† assumed.

## Results

### Using observable model to mimic unobservable model

To analytically describe the difference between two modeling approaches, we consider the unobservable model as a special case of the abovementioned observable model. Figure 1C shows the compartments of a variant of the observable model that are intended to mimic the SEIR structure. To do this, we divide the asymptomatic infected individuals *J*_A_(*t*,*τ*) in Figure 1A into three sub-populations, i.e., (i) pre-symptomatic individuals who are supposed to develop symptom after spending the incubation period, *H*_S_(*t*,*τ*), (ii) asymptomatic non-infectious individuals who will not become symptomatic throughout the course of infection, *H*_A_(*t*,*τ*), and (iii) asymptomatic infectious individuals, *I*(*t*,*τ*). The fate of experiencing symptomatic infection is determined upon infection with a probability *α*, similarly to that taking place when acquiring infectiousness in the SEIR model (Figure 1B). In the following, those who remain asymptomatic throughout the course of infection (i.e. *H*_A_+*I*) is referred to as “fully” asymptomatic, while those who eventually develop symptoms, *H*_S_ is referred to as “pre-symptomatic” for clarity. Recovered individuals at calendar time *t* is denoted by *U*(*t*). The transition rates from *H*_S_ to *J*_S_, *H*_A_ to *I*, *J*_S_ to *U*, and *I* to *U* are *η*(*τ*)/*α*, *ρ*(*τ*), *γ*_S_(*σ*), and *ζ*_A_(*τ*), respectively, where *τ* and *σ* again represent the infection-age and the disease-age, respectively. For consistency between the observable and unobservable models, the transition from *H*_s_ to *J*_s_ is artificially scaled by *α*, because *J*_s_ in the observable model welcomes only the fraction *α* of infected individuals to symptomatic class, which occurs not only during the transition from *H*_s_ to *J*_s_ but also when infected individuals enter to *H*_s_. The time-dependent growth of infected individuals is described by
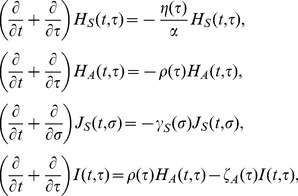
(11)


with the following boundary conditions:
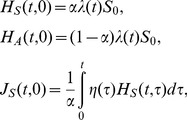
(12)


where the force of infection, *λ*(*t*), is parameterized as
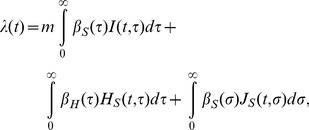
(13)


where *m* is the relative infectiousness among those who remain asymptomatic, *β*_H_ and *β*_S_ are the rates of transmission caused by pre-symptomatic and symptomatic individuals, respectively. It should be noted that *m* is multiplied to only the first integral term, because *m* is defined as the infectiousness of “fully” asymptomatic individuals relative to that among those who experience symptomatic state in the observable model (Figure 1B), as was defined elsewhere [10]. This scaling was required to let the model in Figure 1A mimic the model in Figure 1B. It is evident from Figure 1C that for the unobservable model (Figure 1B) to agree with the observable one (Figure 1C), the incubation period and the latent period must be identical. Moreover, the recovery from an infectious state should also be identical to the recovery from symptomatic illness. Two models become consistent from each other if the following conditions are met:

(a) *α* = *k* (i.e. assumed probabilities of symptomatic infection in two models are identical),

(b) *ε*(*τ*) = *η*(*τ*)/*α* = *ρ*(*τ*) (i.e. the incubation period is identical to the latent period; or equivalently, *β*_H_(*τ*) = 0 for any *τ*),

and (c) *κ*_S_(*τ*) = *γ*_S_(*σ*) and *κ*_A_(*τ*) = *ζ*_A_(*σ*) (i.e., the recovery rates of both models are an identical constant).

Writing in the way we computed the observable model in (4), the basic reproduction number is computed as
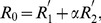
(14)


where
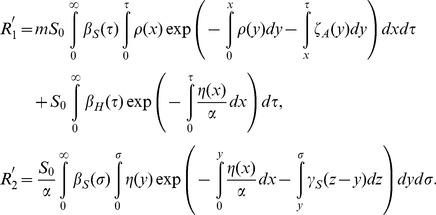
(15)


In summary, two models are rather different and can be consistent only in the case that the model could be written by ordinary differential equations and only when the incubation period can be equated to the latent period.

### Comparison of the basic reproduction number

We continue to compare the special case of the observable model (Figure 1C) with the unobservable SEIR type model (Figure 1B). As was implicated from abovementioned conditions (a)–(c) to ensure consistency between the two models, it should be noted that there is no concept of pre-symptomatic transmission in the unobservable model. On the contrary, the special case (Figure 1C) can still account for pre-symptomatic transmission as long as we assume that *β*_H_(*τ*)>0. Let *g* represent the proportion of pre-symptomatic transmissions among the total of asymptomatic transmissions, then the basic reproduction number of the special case model (14) is rewritten as follows:
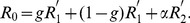
(16)


Let *R*_a_ and *R*_pre_ be the average numbers of secondary cases generated by a single (fully) asymptomatic and pre-symptomatic case, respectively. Using the weighted average of the reproduction numbers with the proportion of symptomatic infections (e.g. as practiced in (9)), *R*_0_ of the model that is intended to bridge the observable model with the unobservable one (Figure 1C) can also be expressed as
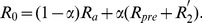
(17)


The average number of secondary cases generated by a single fully asymptomatic case should be identical between (16) and (17), i.e., 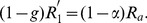
(18)


Similarly, the average number of secondary cases generated by a single pre-symptomatic case should also be identical between the two models as follows:
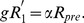
(19)


**Figure 2:**
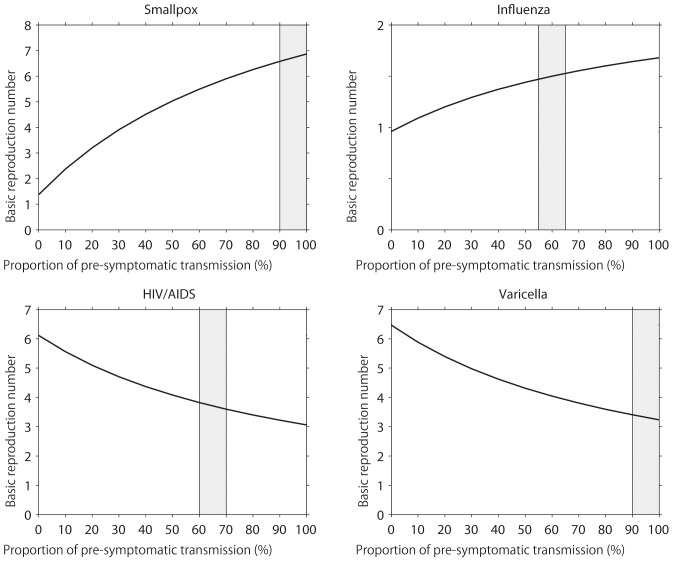
The basic reproduction number and the pre-symptomatic transmission. The impact of varying the proportion of pre-symptomatic transmissions among all asymptomatic transmissions (the horizontal axis; denoted by *g* in the main text) on the basic reproduction number, *R*_0_. Only the value of *g* (and the corresponding *α*) in the model is varied. All other parameters are fixed (see Table? 1). Shaded area represents the plausible parameter region of the proportion of pre-symptomatic transmissions among the total asymptomatic transmissions, *g*, for a specific disease.

Figure 2 examines the impact of *g* on the resulting estimate of the basic reproduction number, varying only *g* (and the corresponding *α*) in the model and using fixed values for all other parameters in equations (16) and (17) (see Table 1). Note that *g* = 0 is the special case in which the observable model (Figure 1C) is fully consistent with the unobservable model. As *g* increases, *R*_0_ for smallpox and influenza are elevated. However, *R*_0_ for HIV and varicella are lowered as a function of *g*. Assuming that *R*_pre_ is proportional to *R*_a_, the differential sensitivity is understood by considering the weighted average in (16) and (17). That is, we have
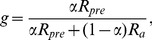
(20)


or *α* = *gR*_a_/{(1−*g*)*R*_pre_+*gR*_a_}, indicating that the larger *g*, the larger *α* has to be. Consequently, if the number of fully asymptomatic transmissions is smaller than other transmissions (in the case of influenza and smallpox), *R*_0_ is an increasing function of *g*. However, when there are substantial pre-symptomatic transmissions (e.g. HIV/AIDS), the relationship between *R*_0_ and *g* is reversed.

### Model building and vaccination

In the following, a comparison of the reproduction numbers under vaccination is made between the observable model (Figure 1A) and the unobservable model (Figure 1B). Because a randomly mixing population is divided into vaccinated and unvaccinated ones, we introduce the next-generation matrix. Let *p*, 1−*q*_S_, 1−*q*_I_, and 1−*q*_D_ be the vaccination coverage, vaccine efficacy in reducing susceptibility, infectiousness, and efficacy of preventing symptomatic illness, respectively. As heuristically derived elsewhere [12], [13], the next-generation matrix that describes secondary transmission between and among vaccinated and unvaccinated cases is employed. Let *ψ*(*τ*) be the so-called reproduction kernel of the renewal process of the observable model that describes the class-age dependent rate of secondary transmission per single infected individual [28], i.e.,
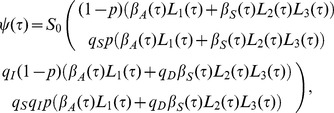
(21)


where the first row represents the exposure to unvaccinated susceptible individuals. It should be noted that *q*_D_ appears inside parenthesis in the second column (i.e. secondary transmissions caused by vaccinated cases). The survival rates *L*_1_(*τ*), *L*_2_(*τ*) and *L*_3_(*τ*) in (21) are written as
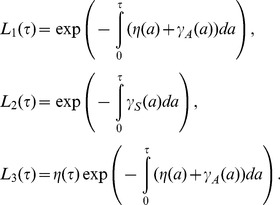
(22)


The next-generation matrix of the observable model under vaccination is given by the integral of *ψ*(*τ*), i.e., 
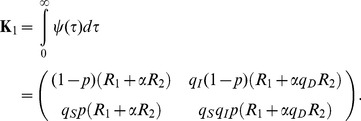
(23)


Let *F*(*σ*) and *L*(*σ*) be matrices that describe the class-age dependent rate of the appearance of new infections and the proportion of those who remain infectious, respectively, i.e., 
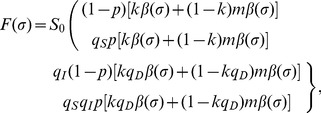
(24)

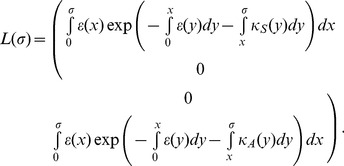
(25)


The next-generation matrix of the unobservable model is obtained from ([28]):
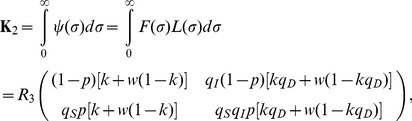
(26)


where *w* is the ratio of *R*_4_ to *R*_3_ and is identical to *m* if *κ*_A_ = *κ*_B_. Note that *q*_D_ only changes the weight of *R*_3_ (or *R*_4_) inside the bracket of all elements. The effective reproduction number is the dominant eigenvalue of these matrices, i.e., 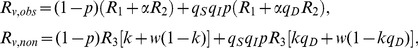
(27)


where *R*_v,obs_ and *R*_v,non_ correspond to the reproduction numbers of the observable and unobservable models, respectively. It should be noted that only *R*_v,obs_ is consistent with the data generating process of *q*_D_, while this is not the case for *R*_v,non_, because *q*_D_ in the equation of *R*_v,non_ is assumed to have had an impact on the transition rate from pre-infectious to infectious period (in addition to the impact on the probability of symptom development alone; Figure 1B).

**Figure 3:**
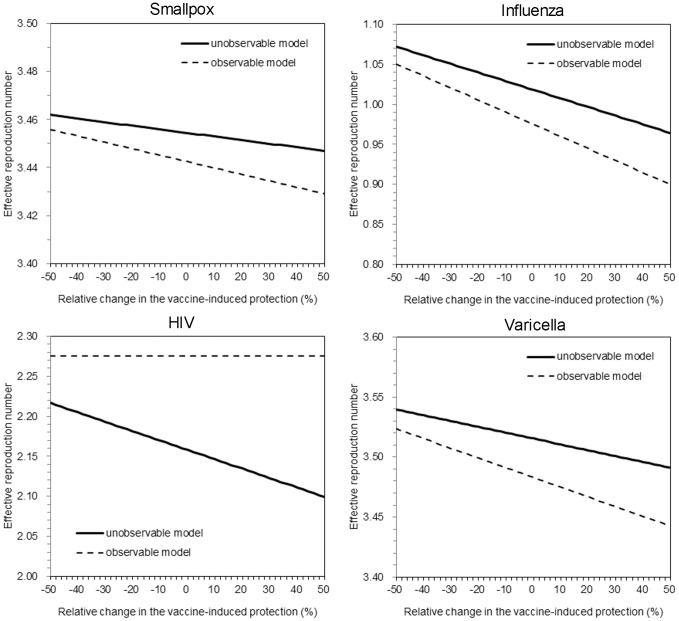
The effective reproduction number under vaccination practice. Effective reproduction numbers for the observable model and the unobservable model are compared as a function of vaccine-induced reduction in symptomatic illness. To permit comparison, in the absence of vaccination practice, the epidemic threshold values of the two models were assumed as identical. Vaccination coverage is fixed at 50%. The solid line shows the reproduction number of the unobservable model under vaccination. The dashed line shows the reproduction number of the observable model under vaccination. Except the vaccine-induced reduction in symptomatic illness, all parameters were fixed (see Table? 1). For the unobservable model, relative infectiousness of asymptomatic individuals (compared to symptomatic individuals), *m* (or *w*), was arbitrarily fixed at 0.5 for three diseases other than varicella to which we assigned 0.7 (these particular values were arbitrarily chosen to visually demonstrate the difference between two models).

To understand the extent of the different impact of *q*_D_ on the reproduction number between two models, Figure 3 compares the values of *R*_v,obs_ and *R*_v,non_ for selected four diseases as a function of vaccine-induced reduction in symptomatic illness, *q*_D_. By varying *q*_D_, different patterns of variation in the reproduction number are seen. For the examined three diseases, i.e., smallpox, influenza and varicella, *R*_v,non_ was greater than *R*_v,obs_. The relationship was reversed for HIV, and in particular, *R*_v,obs_ of HIV was independent of *q*_D_ due to the assumed absence of secondary transmission following the onset of AIDS. Although the difference is subtle for smallpox and varicella, the critical level of influenza is clearly different between two models for influenza. Moreover, it should be noted that the critical coverage is an inverse function of the reproduction number, and a slightly greater reproduction number based on the unobservable model could incorrectly indicate us to vaccinate as many as additional 5–10% of the population as compared to the coverage calculated from the observable model. The difference in the critical coverage was most apparent for HIV/AIDS.

## Discussion

The present study analyzed and compared observable and unobservable modeling approaches. Two major tasks have been completed. First, by rewriting the observable model as if it were an SEIR-type unobservable model, we aimed to clarify underlying assumptions of the unobservable model that involves asymptomatic transmission. For the two models to be identical, we have demonstrated that it is essential that the incubation period has to be identical to the latent period and also that no pre-symptomatic transmission occurs in both models. Only the observable model can directly incorporate vaccine-induced reduction in symptomatic illness (in the manner that the corresponding vaccine effect data is generated), and the probability of symptomatic infection in the unobservable model was shown to be multiplied to the transition rate from pre-infectious to infectious state without phenomenological justification. Second, we numerically solved both models and examined the sensitivity of *R*_0_ to the frequency of pre-symptomatic transmission. We identified that the ignorance of pre-symptomatic transmission in the unobservable model can lead to an overestimate of *R*_0_. Moreover, we have shown that the critical coverage of vaccination can be different between two models, because the vaccine efficacy of preventing symptomatic illness would influence the threshold in different mathematical manners.

The present study emphasizes that an appropriate model formulation would be essential to answer the corresponding scientific or public health question. As we have shown, an explicit formulation would also help clarify underlying assumptions that tend to be hidden in common model structures. Considering a practical example of vaccination that influences the symptom onset, we have shown that the modeling approach to tackle this issue requires a model building approach that can explicitly account for the natural course of infection including asymptomatic and symptomatic states. Since the use of SEIR structure with two or more types of I-classes with different levels of symptom or clinical severity has also partially accounted for this matter of differential severity of symptom, and because the unobservable modeling approach to this issue has been proposed relatively early [10], the similar model structure has become widely adopted in a variety of settings in studying influenza and other directly transmitted infectious diseases [29]–[35]. However, we have shown that the unobservable model has to inherently adopt an assumption that there is no pre-symptomatic transmission, and in this model, vaccine-induced reduction in symptomatic illness has to influence the transition from pre-infectious to infectious state [12]. To explicitly and appropriately incorporate the vaccine effect in reducing the risk of a symptomatic disease into the model, it is fruitful to employ a model that directly accounts for disease progression.

Although our discussion might read as if we regard the observable model as always better than the unobservable one, this preference cannot always be true. In fact, the observable model is not perfect, largely missing the information of infectiousness in the model structure. However, if we handle the model fitting to the incidence of illness onset, the observable model must be most useful, because the renewal equation of only symptomatic cases can be computed and directly fitted to the data [8]. If our study objective was not to quantitatively measure model parameters based on observable empirical data (e.g. model fitting to real data), the unobservable model may be more useful in many other objectives (e.g. in considering the loss of infectiousness during the isolation period). Rather than emphasizing that we should regard the observable model as a default, we would like to emphasize that writing this particular issue from multiple angles would be useful for mathematical modeling studies; the present study was a single study that focused on symptom-based modeling approach in contrast to a classical one. Moreover, it should be noted that “theoretically” the best model in this context would be the one that accounts for both observable and unobservable information within a single model. Such a model can easily address the dependence structure between clinical illness and infectiousness [36], and indeed, the potential dependence and difference between the incubation period and the latent period are known as critical factors in determining the effectiveness of public health interventions including contact tracing and case isolation [9], [37]–[39]. As demonstrated by animal experiments for foot and mouth disease [38], an appropriate combination of well-designed experiments (or observations) and statistical inference could shed light on the scientific approach to (i) considering both illness and infectiousness and (ii) identifying ideal modeling strategy in the future [40].

Four limitations should be noted and described briefly. First, we conducted only univariate sensitivity analysis, ignoring any possible dependence between the frequency of pre-symptomatic transmissions among the total asymptomatic transmissions and other epidemiological variables. Ignoring such dependence structure could sometimes lead to overestimating the effectiveness of public health interventions [41]. Second, we focused on the basic reproduction number, and did not extend epidemiological insights into other important quantities (e.g. growth rate of infections) [42], [43]. Third, to keep the matter as simple as possible, our arguments rested on homogeneously mixing assumptions. Fourth, whereas our model rested on fixed compartment structures (Figure 1), the structure of model ultimately depends on specific diseases and study objectives [44].

Considering that we were successful in gaining useful epidemiological insights into future quantitative modeling by formulating the vaccination issue using an observable model, it is suggested that more studies based on observable epidemiological variables are conducted. Future studies can also tackle the issue of abovementioned dependence between clinical illness and infectiousness based on an explicit model with both pieces of information as variables and analyzing individual datasets with multiple dimensions.
